# New evidences on the altered gut microbiota in autism spectrum disorders

**DOI:** 10.1186/s40168-017-0242-1

**Published:** 2017-02-22

**Authors:** Francesco Strati, Duccio Cavalieri, Davide Albanese, Claudio De Felice, Claudio Donati, Joussef Hayek, Olivier Jousson, Silvia Leoncini, Daniela Renzi, Antonio Calabrò, Carlotta De Filippo

**Affiliations:** 1 Computational Biology Research Unit, Research and Innovation Centre, Fondazione Edmund Mach, Via E. Mach 1, 38010 San Michele all’ Adige, Italy; 2 Centre for Integrative Biology, University of Trento, Via Sommarive 9, 38123 Trento, Italy; 3 Department of Biology, University of Florence, Via Madonna del Piano 6, 50019 Sesto Fiorentino, Florence Italy; 4 Neonatal Intensive Care Unit, Siena University Hospital AOUS, Viale Bracci 16, 53100 Siena, Italy; 5 Child Neuropsychiatry Unit, Siena University Hospital AOUS, Viale Bracci 16, 53100 Siena, Italy; 6 Azienda Unità Sanitaria Locale Umbria 2, Via D. Bramante 37, 05100 Terni, Italy; 7 Department of Experimental and Clinical Biomedical Sciences, Gastroenterology Unit, University of Florence, Viale Morgagni 40, 50139 Florence, Italy; 8 Institute of Agriculture Biology and Biotechnology, National Research Council (CNR), Via Moruzzi 1, 56124 Pisa, Italy

**Keywords:** Autism spectrum disorders, Gut microbiota, Mycobiota, Constipation, Metataxonomy

## Abstract

**Background:**

Autism spectrum disorders (ASDs) are neurodevelopmental conditions characterized by social and behavioural impairments. In addition to neurological symptoms, ASD subjects frequently suffer from gastrointestinal abnormalities, thus implying a role of the gut microbiota in ASD gastrointestinal pathophysiology.

**Results:**

Here, we characterized the bacterial and fungal gut microbiota in a cohort of autistic individuals demonstrating the presence of an altered microbial community structure. A fraction of 90% of the autistic subjects were classified as severe ASDs. We found a significant increase in the *Firmicutes*/*Bacteroidetes* ratio in autistic subjects due to a reduction of the *Bacteroidetes* relative abundance. At the genus level, we observed a decrease in the relative abundance of *Alistipes*, *Bilophila*, *Dialister*, *Parabacteroides*, and *Veillonella* in the ASD cohort, while *Collinsella*, *Corynebacterium*, *Dorea*, and *Lactobacillus* were significantly increased. Constipation has been then associated with different bacterial patterns in autistic and neurotypical subjects, with constipated autistic individuals characterized by high levels of bacterial taxa belonging to *Escherichia/Shigella* and *Clostridium cluster XVIII*. We also observed that the relative abundance of the fungal genus *Candida* was more than double in the autistic than neurotypical subjects, yet due to a larger dispersion of values, this difference was only partially significant.

**Conclusions:**

The finding that, besides the bacterial gut microbiota, also the gut mycobiota contributes to the alteration of the intestinal microbial community structure in ASDs opens the possibility for new potential intervention strategies aimed at the relief of gastrointestinal symptoms in ASDs.

**Electronic supplementary material:**

The online version of this article (doi:10.1186/s40168-017-0242-1) contains supplementary material, which is available to authorized users.

## Background

The term “autism spectrum disorders” (ASDs) refers to a group of neurodevelopmental disorders with an early life stage onset characterized by alterations in social interactions and communication and by restricted and repetitive behaviour [1]. It is now well accepted the contribution of both genetic and environmental factors in the aetiology of ASDs [2, 3]. Among the non-neurological symptoms associated with ASDs, several studies indicate gastrointestinal (GI) symptoms as common comorbidities [4–7]. Alterations in the composition of the gut microbiota have been implicated in a wide variety of human diseases, including ASDs [8]. Since the gut microbiota makes critical contributions to metabolism and maintenance of immune homeostasis and may control the central nervous system (CNS) activities through neural, endocrine, and immune pathways [9], it has been hypothesized the active role of the gut microbiota in ASD pathophysiology. There is more than a subtle link between the gut microbiota and the CNS, through the so-called “microbiome-gut-brain axis”. Indeed, it has been demonstrated a direct interaction between the gut microbiota and enteric neurons [10, 11], its role in the regulation of the HPA axis [12], and the production of many chemicals important in brain functioning (e.g*.*, serotonin, dopamine, kynurenine, *γ*-aminobutyric acid, SCFAs, *p*-cresol) [13, 14]. A dysbiotic microbial community could lead to systemic inflammation due to hyper-activation of T-helper 1 and T-helper 17 cell responses [15] affecting also the reactivity of peripheral immune cells to the CNS [16] and the integrity of blood-brain barrier [17] which is known to be altered in ASDs [18]. Several evidences suggested an early immune activation with chronic inflammation and cytokine dysregulation in ASDs [19, 20], and it has been shown that systemic inflammation induced by LPS provokes behavioural changes and impairs the blood-brain barrier in animal models [17, 21]. Furthermore, fungal infections that may originate from alterations in commensal bacteria population [22] could shift the indoleamine 2,3-dioxygenase’s activity [23, 24] reducing the levels of kynurenine [25], a neuroprotective agent. Despite several reports disclosed an aberrant gut microbiota in ASDs, consensus across studies has not yet been established [8]. Here, we characterized the bacterial gut microbiota and the less studied gut mycobiota of subjects affected by autism through amplicon-based metataxonomic analysis of the V3–V5 regions of the prokaryotic 16S ribosomal DNA and of the internal transcribed spacer 1 (ITS1) region of the fungal rDNA in order to better understand the microbial community structure associated with ASDs and its involvement on GI abnormalities.

## Results

### Autistic subjects harbour an altered bacterial gut microbiota

**Fig. 1:**
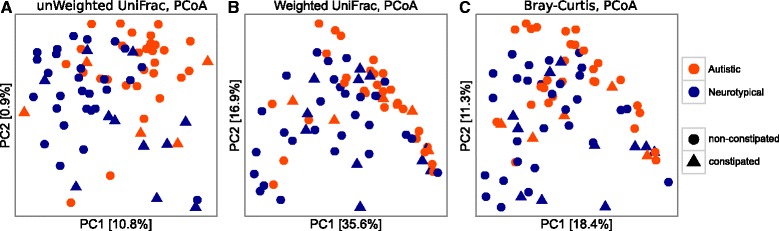
PCoA of bacterial beta diversity based on the **a** unweighted and **b** weighted UniFrac distances and **c** Bray-Curtis dissimilarity. Autistic and neurotypical subjects are coloured in *orange* and *blue*, respectively. The constipation status of the subjects is indicated according to different shapes, *circles* for non-constipated and *triangles* for constipated individuals

For the characterization of the gut microbiota associated with autism, we recruited 40 autistic subjects (36 out of 40 autistic subjects were classified as severe ASDs, Childhood Autism Rating Scale (CARS) value >37) and 40 neurotypical controls (Table 1, Additional file 1: Table S1). Analysis of *alpha* diversity revealed no significant differences between autistic and neurotypical subjects (hereinafter termed AD and NT, respectively). However, the analysis of the *beta* diversity calculated on the unweighted and weighted UniFrac distances and the Bray-Curtis dissimilarity revealed that the bacterial microbiota of AD clusters apart from that of NT (*p* < 0.05, permutational multivariate analysis of variance (PERMANOVA); Fig. 1, Additional file 2: Table S2). Since we enrolled subjects suffering from constipation among NT and AD subjects, the impact of constipation on the beta diversity of the two groups of study was also analysed. We observed that constipation has a significant effect on the microbial community structure within NT subjects (*p* < 0.05, PERMANOVA), as expected [26, 27], but not within AD subjects (Additional file 2: Table S2). Furthermore, we observed that the severity of the autistic phenotype, as measured by CARS scores, does not affect the bacterial community structure among AD individuals (*p* > 0.05, PERMANOVA; Additional file 3: Table S3).

**Table 1:** Characteristics of study participants

	Autistic	Neurotypical
Subjects (*n*)	40	40
Age (1st–3rd quartile)	10 (5–17)	7 (3.6–12)
Gender (*n*)		
Female	22.5% (9)	30% (12)
Male	77.5% (31)	70% (28)
Constipation (*n*)		
Constipated	12.5% (5)	27.5% (11)
Non-constipated	72.5% (29)	72.5% (29)
NA	15% (6)	0% (0)
Calprotectin (1st–3rd quartile)	36.9 (17.6–76.0) μg/g	40.9 (17–74.7) μg/g
Constipated	39.1 (22.9–70.0) μg/g	27.9 (20.3–97.6) μg/g
Non-constipated	35.9 (15.0–57.8) μg/g	50.5 (15.0–73.8) μg/g
CARS (1st–3rd quartile)	47 (40–50.5)	NA
Constipated	50 (36–52.0)	NA
Non-constipated	48 (42–50.0)	NA
ESR (1st–3rd quartile)	7.5 (3.25–17.7) mm/h	NA
Constipated	22.0 (12.0–25.0) mm/h	NA
Non-constipated	7.0 (2.7–11.2) mm/h	NA
Serum IgA (1st–3rd quartile)	131.0 (70.0–172.2) mg/ml	NA
Constipated	97.0 (82.0–153.0) mg/ml	NA
Non-constipated	133.0 (67.0–181.0) mg/ml	NA

Data expressed as medians with interquartile ranges when applicable. *AD* autistic subjects, *NT* neurotypical subjects, *NA* not applicable, *CARS* Childhood Autism Rating Scale, *ESR* erythrocyte sedimentation rate

**Fig. 2:**
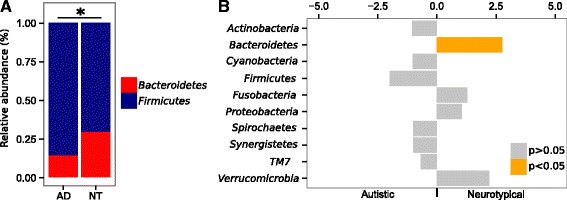
**a** Mean relative abundances (%) of *Firmicutes* and *Bacteroidetes* in autistic (AD) and neurotypical (NT) subjects; *p < 0.005, Wilcoxon rank-sum test on the *Firmicutes*/*Bacteroidetes* ratio. **b** Welch’s *t* test statistics of the relative abundances of bacterial phyla in autistic and neurotypical subjects. *Orange bars* indicate significant FDR-corrected *p* values adjusted for multiple comparison controlling the family-wise type I error rate

**Fig. 3:**
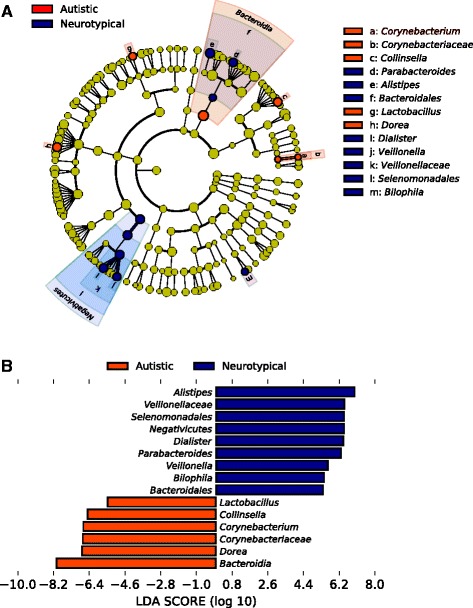
**a** Cladograms generated by LEfSe indicating differences in the bacterial taxa between autistic (AD) and neurotypical (NT) subjects. Nodes in *orange* indicate taxa that were enriched in AD compared to those in NT, while nodes in *blue* indicate taxa that were enriched in NT compared to those in AD. **b** LDA scores for the bacterial taxa differentially abundant between autistic (AD) and neurotypical (NT) subjects. Positive and negative LDA scores indicate the bacterial taxa enriched in NT and AD subjects, respectively. Only the taxa having a *p* < 0.01 (Wilcoxon rank-sum test) and LDA >2.0 are shown in the figure legend

Phylum level analysis showed a clear alteration of the bacterial gut community in AD characterized by a higher *Firmicutes*/*Bacteroidetes* ratio (*p* < 0.005, Wilcoxon rank-sum test; Fig. 2a) in AD than that in NT due to a significant reduction of the relative abundance of *Bacteroidetes* (9.2% AD, 19.4% NT) (FDR-corrected *p* < 0.05, Welch *t* test; Fig. 2b). Genus level analysis showed that the top ten most abundant genera in both AD and NT subjects were *Bifidobacterium*, *Bacteroides*, *Faecalibacterium*, *Unknown Lachnospiraceae*, *Blautia*, *Ruminococcus*, *Clostridium XI*, *Streptococcus*, *Gemmiger*, and *Lachnospiraceae incertae sedis* (Additional file 4: Figure S1, Additional file 5: Table S4). Interestingly, the genus *Prevotella* was only barely represented in AD with respect to NT (0.05% AD, 1.5% NT), in agreement with a previous study on the gut microbiota in ASD children [28] although this difference of relative abundance was not supported by the statistical analysis. We further analysed the bacterial community structure associated with AD and NT by using linear discriminant effect size (LEfSe), an algorithm for high-dimensional biomarker discovery which uses linear discriminant analysis (LDA) to estimate the effect size of each taxon which is differentially represented in cases and controls [29]. LEfSe analysis revealed a significant increase of the relative abundance of different bacterial taxa in AD than in NT among which *Collinsella*, *Corynebacterium*, *Dorea*, and *Lactobacillus* and a significant reduction of the taxa *Alistipes*, *Bilophila*, *Dialister*, *Parabacteroides*, and *Veillonella* in AD than in NT (*p* < 0.01, Wilcoxon rank-sum test; LDA >2.0; Fig. 3).

### Constipation selects different bacterial taxa in autistic subjects and neurotypical healthy controls

**Fig. 4:**
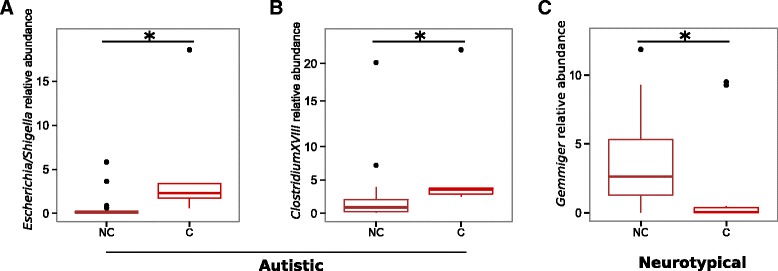
Box plot representation of the relative abundances of bacterial genera correlating with the constipation status of the subjects enrolled in this study. Comparisons between **a** and **b** constipated (C) and non-constipated (NC) autistic subjects and (**c**) constipated (C) and non-constipated (NC) neurotypical subjects; *Asterisk* indicates FDR-corrected *p* < 0.05, Wilcoxon rank-sum test

Autistic subjects frequently suffer of GI comorbidities [4–7], and constipation is a GI symptom often reported in these subjects, known to alter the physiology of the human GI tract and the gut microbiota itself [27, 30, 31]. Correlation analysis of the bacterial relative abundances between constipated and non-constipated subjects, both autistic and neurotypical, revealed that among the most abundant bacterial genera (with relative abundance >0.5% and detectable in at least the 70% of the investigated subjects), the taxa *Gemmiger* and *Ruminococcus* anticorrelates with the constipation status (Spearman’s correlation *r* = −0.39 and −0.36, respectively; FDR-corrected *p* < 0.05; Additional file 6: Table S5) while *Escherichia/Shigella* and *Clostridium cluster XVIII* positively correlates with this GI symptom (Spearman’s correlation *r* = 0.31 and 0.38, respectively; FDR-corrected *p* < 0.05; Additional file 6: Table S5). We further compared the relative abundance of these taxa among constipated and non-constipated subjects within and between groups. We observed that *Escherichia/Shigella* and *Clostridium cluster XVIII* were significantly more abundant in constipated AD compared to the non-constipated ones (FDR-corrected *p* < 0.05, Wilcoxon rank-sum test; Fig. 4a, b) while no differences have been detected between constipated and non-constipated NT for these taxa. On the other hand, the genus *Gemmiger* was significantly less abundant in constipated compared to non-constipated NT (FDR-corrected *p* < 0.05, Wilcoxon rank-sum test; Fig. 4c). Remarkably, no significant differences have been observed in the levels of faecal calprotectin between AD and NT as well as between constipated and non-constipated subjects in both groups (Table 1 and Additional file 1: Table S1). Furthermore, we analysed the levels of other two biomarkers of inflammations, i.e*.*, serum IgA and ESR in the autistic subjects, and we did not observe significant differences among constipated and non-constipated AD (Table 1 and Additional file 1: Table S1). Therefore, while constipation resulted in a significant increase of *Escherichia/Shigella* and *Clostridium cluster XVIII*, no differences have been observed in the levels of inflammation between constipated and non-constipated autistic subjects suggesting that constipation and the related alterations of the gut microbiota in autistic subjects as well as in neurotypical individuals are not associated with an increase of intestinal inflammation. It should be noted that the number of enrolled constipated subjects was quite low and therefore these analyses could be underpowered.

### Autistic subjects harbour an altered gut mycobiota

**Fig. 5:**
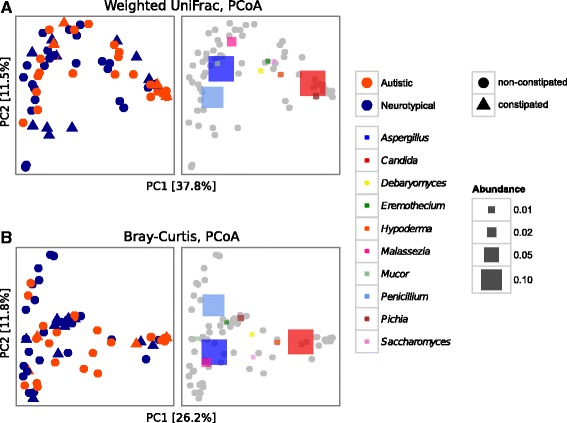
PCoAs of fungal beta diversity based on **a** weighted UniFrac distance and **b** Bray-Curtis dissimilarity. The right panel of the graphs **a** and **b** shows the same PCoA coordinates with the most abundant OTUs superimposed as *coloured squares*, with the size being proportional to the mean relative abundance of the taxon across all samples (*grey dots*). Autistic and neurotypical subjects are colored in *orange* and *blue*, respectively. The constipation status of the subjects is indicated according to different shapes, *circles* for non-constipated and *triangles* for constipated individuals

We then investigated the gut mycobiota of our study cohort through amplicon-based sequencing of fungal ITS1 region. High-quality fungal sequences were detected respectively in 35 out of 40 autistic subjects and 38 out of 40 NT. As occurred for the bacterial gut microbiota, we did not observe significant differences in fungal alpha diversity between AD and NT. The analysis of beta diversity revealed that the gut mycobiota of AD was different compared to NT as calculated by principal coordinates analysis (PCoA) and PERMANOVA on the weighted UniFrac distance and Bray-Curtis dissimilarity (*p* < 0.05; Fig. 5). As for the bacterial beta diversity, constipation showed a significant effect within NT subjects (*p* = 0.046, PERMANOVA on Bray-Curtis dissimilarities) but not within AD subjects (Additional file 7: Table S6). Furthermore, the severity of the autistic phenotype does not affect the gut mycobiota community structure among AD individuals (*p* > 0.05, PERMANOVA; Additional file 3: Table S3). An in-depth analysis of the gut mycobiota leads to the identification of 50 fungal taxa fully classified to the genus level and 30 only partially classified. Genus level analysis showed *Aspergillus* (24.2% AD; 28% NT), *Candida* (37.7% AD; 14.1% NT), *Penicillium* (13.2% AD; 23.5% NT) and *Malassezia* (3.05% AD; 3.3% NT) as the most abundant and widely distributed genera in our study cohort in terms of relative abundance (Additional file 8: Figure S2, Additional file 9: Table S7). The relative abundance of the genus *Candida* was more than twice as much in AD than NT, yet due to a large dispersion of values (*p* < 0.001; Levene’s test), this difference was only partially significant (Welch *t* test, FDR-corrected *p value* = 0.09, uncorrected *p value* = 0.006; Additional file 10: Figure S3). The superimposition of the most abundant genera over the PCoA plots revealed that high levels of *Candida* abundance was associated with a group of subjects mainly affected by autism (Fig. 5) suggesting that *Candida* indeed could play a role in the altered microbial community associated with the autistic subjects. Correlation analyses among the most abundant fungi and bacteria (with relative abundance >0.5% and detectable in at least the 70% of the investigated subjects) revealed no significant correlations among autistic subjects while a significant positive correlation between the genera *Aspergillus* and *Bifidobacterium* was found within neurotypical individuals (Spearman’s *r* = 0.6, FDR-corrected *p* = 0.004) (Additional file 11: Table S8).

## Discussion

The gut microbiota is a crucial factor for the maintenance of the GI tract functions and immune homeostasis. It is well known that dysbiosis of the GI tract could lead to inflammation and immune activation in several pathologies [15]. The frequent occurrence of GI symptoms in autistic subjects imply the possible involvement of the gut microbiota in ASD gastrointestinal pathophysiology, further supported by the speculations on the increased incidence of ASD cases due to “Western” habits (i.e., diet, medications, and excessive overall hygiene) that can affect the composition of the gut microbiota [32]. Several studies demonstrated alterations in the bacterial gut microbiota of ASD individuals, even if the differences reported in these studies were in some cases discordant, possibly due to variance in sampling strategies and methodologies used [8]. In addition, our recent findings showed an altered gut microbiota in Rett syndrome [33], a genetically determined neurodevelopmental disorder previously categorized in the ASD group sharing some features of these conditions. We characterized the gut microbiota associated with autism, disclosing an altered microbial community both at bacterial and fungal level. We observed a significant increase in the *Firmicutes*/*Bacteroidetes* ratio in autistic subjects due to a significant reduction of *Bacteroidetes* in these individuals. Several inflammatory conditions have been related to an increase in the *Firmicutes*/*Bacteroidetes* ratio such as inflammatory bowel diseases (IBDs) [34] and obesity [35]. Consistently with these observations, an increased *Firmicutes*/*Bacteroidetes* ratio has been reported also in subjects with autism [36, 37]. Furthermore, we discovered that the relative abundances of the genera *Collinsella*, *Corynebacterium*, *Dorea*, and *Lactobacillus* were significantly increased in the gut microbiota of autistic subjects with respect to that of the neurotypical subjects while the relative abundance of the genera *Alistipes*, *Bilophila*, *Dialister*, *Parabacteroides*, and *Veillonella* were significantly reduced in these individuals. A recent study on a mouse model of ASDs demonstrated that treatments with a PSA^+^ *Bacteroides fragilis* strain restore autism-related behavioural and GI abnormalities, also reducing the reported high levels of *Lachnospiraceae* and 4-ethylphenyl sulfate, a metabolite produced by this bacterial family related to *p*-cresol, a putative metabolic marker for autism [38]. Overall, these data are consistent with our findings and remark the importance of *Bacteroidetes* in ASD pathophysiology. Moreover, *Lactobacillus* resulted to be enriched in the gut microbiota of autistic individuals while *Dialister* and *Veillonella* resulted to be depleted, in line with the results obtained in previous studies [28, 39]. Since constipation is a common gastrointestinal problem in subjects with ASDs [4–7], we compared our data between constipated and non-constipated subjects in order to evaluate the contribution of constipation in shaping the gut microbiota of autistic subjects. Indeed, it has been proposed that GI symptoms may be related to ASDs [40]. The evidence that the taxa belonging to the *Clostridium cluster XVIII* and the putative pro-inflammatory *Escherichia/Shigella* [41, 42] positively correlated with the constipation status of the subjects as well as their enrichment in constipated autistic subjects supports the hypothesis that GI problems and related alterations of the gut microbiota may contribute to ASD gastrointestinal symptoms [40]. Because of their ability to produce exotoxins and propionate that may exacerbate autistic symptoms [43], the role of clostridia in ASDs has been extensively explored. The species belonging to the *Clostridium cluster XVIII* have been shown to produce exotoxins [44] and to promote conditions favouring inflammation [45, 46] although other studies observed their potential ability to induce homeostatic T-reg responses [47]. It is also interesting to underline the occurrence of a subclinical acute phase response in ASD plasma, as evidenced by advanced proteomic analysis [48].

Despite the importance given to the implications of the gut microbiota in health and disease, few reports have explored the relevance of the fungal component of the gut microbiota in GI (patho) physiology [49]. Furthermore, none of the published studies on ASDs’ gut microbiota have assessed the fungal gut community structure associated with autism. Our dataset of autistic subjects displayed a different fungal community structure compared to neurotypical subjects. In particular, the genus *Candida* was one of the most abundant taxa in the gut mycobiota of this study cohort, being two times more abundant in AD than in NT. To the best of our knowledge, this is the first time that alterations of the intestinal fungal microbiota are associated with ASDs. Although *Candida* is one of the most common and abundant genus of the human gut mycobiota [50, 51], its implication in phenomena of fungal dysbiosis have been reported in several GI and inflammatory conditions [52–54] as well as in Rett syndrome [33]. It is therefore possible that alteration of the intestinal fungal population driven by an expansion of *Candida* in the gut mycobiota of autistic individuals may negatively impact on GI abnormalities through cytokine dysregulation. The gut microbiota, in particular some species of *Lactobacillus*, modulates the immunological responses to *Candida* in the GI tract by providing tryptophan-derived aryl hydrocarbon receptor ligands that stimulate the immune system, principally ILC3 cells, to produce IL-22 [55]. Together with IL-17, IL-22 avoids the excessive proliferation of *Candida* and other fungal commensals in the gut. It is therefore possible that alterations of the gut microbiota in ASDs could lead to an expansion of the *Candida* population preventing from full restoration of the bacterial community structure. Indeed, it has been observed that alterations of the bacterial gut microbiota due to prolonged antibiotic usage and the subsequent colonization with *C. albicans* interfere with the reassembly of the bacterial community structure, resulting in altered abundances of *Bacteriodetes*, *Lactobacillaceae*, *Ruminococcaceae*, and *Lachnospiraceae* [56]. Since the two different microbial communities (fungi and bacteria) mutually influence, we could also speculate that the reduced early life encounters with foodborne and environmental bacteria and fungi in urban areas of the globalized world could be a cause of the increased colonization with some major commensals, such as the pathobionts *Candida* and *Escherichia*. The alteration of the composition of the gut microbiota could also be mediated by mechanism of trained immunity [57] or by a reduced ability of the immune system to control its overgrowth due to lack of immune training, extending the hygiene hypothesis [58] from bacteria to yeasts [59].

## Conclusions

Here, we observed an altered intestinal microbial community associated with ASDs, both at bacterial and fungal level not depending by the constipation status of autistic individuals but rather by the autistic disorder itself. However, due to the broad phenotypical variability of ASDs, an in-depth characterization of the genetic and phenotypical background in a larger cohort of ASD individuals would be necessary to comprehensively understand the role of the gut microbiota in ASDs pathophysiology and to further validate these findings. Our results therefore encourage new extensive, multicentric studies on the impact of the bacterial and fungal components of the gut microbiota in the gastrointestinal physiology and neuroplastic changes in ASDs, as well as the integration of such data with genetics, immunology, and metabolomics to further establish the relevance of the gut microbiota in the ASDs.

## Methods

### Study participants and samples’ handling and collection

We recruited 40 subjects with clinical diagnosis of autism (average age 11.1 ± 6.8; sex, male:female, 31:9) and we compared them with 40 age and sex-matched neurotypical healthy subjects (average age 9.2 ± 7.9; sex, male:female, 28:12). Autistic subjects with clinically evident inflammatory conditions were excluded. Constipation and inflammation (i.e., serum IgA, erythrocyte sedimentation rate, and faecal calprotectin levels) were also assessed. The autistic subjects were consecutively admitted to the Child Neuropsychiatry Unit of the University Hospital of Siena, and ASDs were diagnosed according with the Diagnostic and Statistical Manual of Mental Disorders, 5th Edition [1], and evaluated using Autism Diagnostic Observation Schedule and Autism Behaviour Checklist. Childhood Autism Rating Scale (CARS) scores [60] were calculated by an experienced child neuropsychiatrist. Average CARS values were 46.2 ± 6.8 (value range 32–57); a fraction of 90% (36/40) were classified as severe ASDs (CARS value >37), with 10% (4/40) being moderately severe ASDs (CARS values from 30 to 36) (Additional file 12: Table S9). No specific comorbidities in the autistic cohort were present with the single exception of a coexisting celiac disease in two patients (5%).

Constipation has been defined according to Rome III criteria [61]. Stool samples from enrolled subjects were collected, aliquoted as it is, and stored at −80 °C until analysis. All subjects of this study were under a Mediterranean-based diet, and no antibiotics, probiotics, or prebiotics have been taken in the 3 months prior to the sample collection. None of the subjects were on anti-inflammatory or antioxidant drugs. The study was conducted after the approval by the Institutional Review Board of the Siena University Hospital (AOUS, Siena, Italy) and all written informed consents were obtained from either the parents or the legal tutors of the enrolled subjects, in compliance with national legislation and the Code of Ethical Principles for Medical Research Involving Human Subjects of the World Medical Association (Declaration of Helsinki).

### Faecal calprotectin assay

Calprotectin determination was performed by using a polyclonal antibody in an enzyme-linked immunosorbent assay (Calprest, Eurospital, Trieste, Italy) according to the manufacturer’s instructions. Calprotectin values <50 μg/g per stool sample were considered normal.

### Pyrosequencing and data analysis

Total DNA extraction from faecal samples (250 mg, wet weight) was performed using the FastDNA™ SPIN Kit for Feces (MP Biomedicals, Santa Ana, CA, USA) following manufacturer’s instructions. For each DNA sample, we amplified respectively the bacterial 16S rRNA genes using a primer set specific for V3–V5 hypervariable regions (F357: 5′-TCCTACGGGAGGCAGCAG-3′ and R937: 5′-TGTGCGGGCCCCCGTCAATT-3′) and the internal transcribed spacer (ITS) using a primer set specific for fungal ITS1 rDNA region (18SF: 5′-GTAAAAGTCGTAACAAGGTTTC-3′ and 5.8S1R: 5′-GTTCAAAGAYTCGATGATTCAC-3′) [62] containing adaptors, key sequence, and barcode sequences as described by the 454 Sequencing System Guidelines for Amplicon Experimental Design (Roche, Basel, Switzerland). The PCR products obtained were then purified, quantified, and pooled in equimolar way in a final amplicon library. The 454 pyrosequencing was carried out on the GS FLX+ system using the XL+ chemistry following the manufacturer’s recommendations (Roche, Basel, Switzerland). Raw 454 data were demultiplexed using the Roche’s sff file software and submitted to the European Nucleotide Archive (ENA) with accession numbers PRJEB15418 and PRJEB15420. Sample accessions IDs and metadata are available in Additional file 12: Table S9. Reads were preprocessed using the MICCA pipeline (v. 0.1) (http://www.micca.org/) [63]. Operational taxonomic units (OTUs) were assigned by clustering the sequences with a threshold of 97% pairwise identity andv their representative sequences were classified using the RDP classifier version 2.7 on 16S data and using the RDP classifier version 2.8 [64] against the UNITE fungal ITS database [65] on ITS1 data. Template-guided multiple sequence alignment (MSA) was performed using PyNAST [66] (v. 0.1) against the multiple alignment of the Greengenes [67] database (release 13_05) filtered at 97% similarity for bacterial sequences and through de novo MSA using T-Coffee [68] for fungal sequences. Fungal taxonomy assignments were then manually curated using BLASTn against the GenBank’s database for accuracy. High-quality fungal sequences have been also manually filtered out for sequences belonging to *Agaricomycetes* (unlikely to be residents of the human gut due to their ecology [69]). The phylogenetic tree was inferred using micca-phylogeny [70]. Sampling heterogeneity was reduced by rarefaction. Alpha (within-sample richness) and beta-diversity (between-sample dissimilarity) estimates were computed using the phyloseq R package [71]. PERMANOVA test was performed using the adonis() function in the R package vegan with 999 permutations. Permutations have been constrained within age groups (corresponding to 0–2, 3–10, 11–17, and >18 years old) or gender to evaluate possible biases related to the unequal age and gender distributions among subjects using the “strata” argument within the adonis() function. Two-sided, unpaired Welch *t* statistics were computed using the function mt() in the phyloseq library and the *p* values were adjusted for multiple comparisons controlling the family-wise type I error rate (minP procedure) [72]. Spearman’s correlation tests were computed using the *psych* R package [73]. Linear discriminant effect size (LEfSe) analysis was performed to find features (taxa) differentially represented between autistic and neurotypical subjects. LEfSe combines Kruskal-Wallis test or pairwise Wilcoxon rank-sum test with linear discriminant analysis (LDA). It ranks features by effect size, which put features that explain most of the biological difference at top. LEfSe analysis was performed under the following conditions: α value for the statistical test equal to 0.01 and threshold on the logarithmic LDA score for discriminative features equal to 2.0 [29]. All statistical analyses were performed using R [74], and *p* values were FDR corrected [75].

## Additional files

Additional file 1: Table S1. Statistical comparisons (Wilcoxon rank-sum test) of clinical data among autistic (AD) and neurotypical (NT) subjects both constipated (C) and non-constipated (NC). (PDF 85 kb)
Additional file 2: Table S2. Permutational multivariate analysis of variance (PERMANOVA) tests of the bacterial gut microbiota on the unweighted and weighted UniFrac distances and the Bray-Curtis dissimilarity according to individuals’ health status and constipation. (PDF 160 kb)
Additional file 3: Table S3. Permutational multivariate analysis of variance (PERMANOVA) tests of the bacterial and fungal gut microbiota on the unweighted and weighted UniFrac distances and the Bray-Curtis dissimilarity according to the severity of the autistic phenotype. (PDF 11 kb)
Additional file 4: Figure S1. Relative abundances at the genus level of the bacterial gut microbiota of autistic (AD) and neurotypical (NT) subjects both constipated (C) and non-constipated (NC). (PDF 1830 kb)
Additional file 5: Table S4. Mean relative abundance (%) ± standard deviation (SD) of bacterial taxa at genus levels in autistic (AD) subjects and neurotypical (NT) controls subjects both constipated (C) and non-constipated (NC). (PDF 303 kb)
Additional file 6: Spearman’s correlation analysis among the most abundant bacterial genera and the constipation status of the subjects of the study cohort. (PDF 154 kb)
Additional file 7: Table S6. Permutational multivariate analysis of variance (PERMANOVA) tests of the fungal gut microbiota on the unweighted and weighted UniFrac distances and the Bray-Curtis dissimilarity according to individuals’ health status and constipation. (PDF 159 kb)
Additional file 8: Figure S2. Relative abundances at the genus level of the fungal gut microbiota of autistic (AD) and neurotypical (NT) subjects both constipated (C) and non-constipated (NC). (PDF 346 kb)
Additional file 9: Table S7. Mean relative abundance (%) ± standard deviation (SD) of fungal taxa at genus levels in autistic (AD) subjects and neurotypical (NT) controls subjects both constipated (C) and non-constipated (NC). (PDF 199 kb)
Additional file 10: Figure S3.
*Candida* relative abundance in autistic (AD) and neurotypical (NT) subjects. *Candida* relative abundances are reported as mean ± standard error. (PDF 19 kb)
Additional file 11: Table S8. Spearman’s correlation analysis among the most abundant bacterial genera and fungal genera in autistic and neurotypical subjects. (PDF 253 kb)
Additional file 12: Table S9. Correspondences among deposited metagenomic data and samples. (XLS 70 kb)

## Abbreviations

AD: Autistic subjects
ASDs: Autism spectrum disorders
CARS: Childhood Autism Rating Scale
CNS: Central nervous system
ESR: Erythrocyte sedimentation rate
FDR: False discovery rate
GI: Gastrointestinal
IBD: Inflammatory bowel disease
ITS: Internal transcribed spacer
NT: Neurotypical subjects
OTU: Operational taxonomic unit
PCoA: Principal coordinates analysis
PERMANOVA: Permutational multivariate analysis of variance
SCFAs: Short chain fatty acids
