# Supplementary material for: New evidences on the altered gut microbiota in autism spectrum disorders
Source: Microbiome. 2017 Feb 22;5:24. doi: 10.1186/s40168-017-0242-1 (PMC5320696; doi:10.1186/s40168-017-0242-1)
Supplement: Additional file 1: Table S1. — Statistical comparisons (Wilcoxon rank-sum test) of clinical data among autistic (AD) and neurotypical (NT) subjects both constipated (C) and non-constipated (NC). (PDF 85 kb) [file 40168_2017_242_MOESM1_ESM.pdf]

Supplementary Table 1: Statistical comparisons (Wilcoxon rank sum test) of clinical data among autistic (AD) and neurotypical (NT) subjects both constipated (C) and non-constipated (NC).

	AD vs NT	AD-C vs NT-C	AD-NC vs NT-NC	AD-C vs AD-NC	NT-C vs NT-NC
**Age**	0.14	0.20	0.64	0.88	0.10
**Calprotectin**	0.75	0.94	0.53	0.56	0.68
**CARS**	NA	NA	NA	0.98	NA
**ESR**	NA	NA	NA	0.98	NA
**Serum IgA**	NA	NA	NA	0.45	NA
