# Supplementary material for: New evidences on the altered gut microbiota in autism spectrum disorders
Source: Microbiome. 2017 Feb 22;5:24. doi: 10.1186/s40168-017-0242-1 (PMC5320696; doi:10.1186/s40168-017-0242-1)
Supplement: Additional file 2: Table S2. — Permutational multivariate analysis of variance (PERMANOVA) tests of the bacterial gut microbiota on the unweighted and weighted UniFrac distances and the Bray-Curtis dissimilarity according to individuals’ health status and constipation. (PDF 160 kb) [file 40168_2017_242_MOESM2_ESM.pdf]

Supplementary Table 2: Permutational multivariate analysis of variance (PERMANOVA) tests of the bacterial gut microbiota on the unweighted and weighted UniFrac distances and the Bray-Curtis dissimilarity according to individuals' health status and constipation.

	Metric	F	R^2	*p-value*
**NT** (n=40) *vs* **AD** (n=40)	Unweighted Unifrac	2.31	0.02	0.001
	Weighted Unifrac	3.29	0.04	0.010
	Bray-Curtis	2.35	0.02	0.005
**AD-C** (n=5) *vs* **AD-NC** (n=29)	Unweighted Unifrac	1.36	0.04	0.058
	Weighted Unifrac	0.98	0.03	0.397
	Bray-Curtis	1.23	0.03	0.226
**NT-C** (n=11) *vs* **NT-NC** (n=29)	Unweighted Unifrac	2.21	0.05	0.001
	Weighted Unifrac	2.13	0.05	0.048
	Bray-Curtis	1.81	0.04	0.030
**AD-C** (n=5) *vs* **NT-C** (n=11)	Unweighted Unifrac	0.44	0.03	0.991
	Weighted Unifrac	0.37	0.02	0.925
	Bray-Curtis	0.44	0.03	0.989
**AD-NC** (n=29) *vs* **NT-NC** (n=29)	Unweighted Unifrac	3.10	0.05	0.001
	Weighted Unifrac	4.49	0.07	0.002
	Bray-Curtis	2.93	0.04	0.002

NT, neurotypical subjects; AD, autistic subjects; NT-C, constipated neurotypical subjects; NT-NC, non-constipated neurotypical subjects; AD-C, constipated autistic subjects; AD-NC, non-constipated autistic subjects.
