# Supplementary material for: New evidences on the altered gut microbiota in autism spectrum disorders
Source: Microbiome. 2017 Feb 22;5:24. doi: 10.1186/s40168-017-0242-1 (PMC5320696; doi:10.1186/s40168-017-0242-1)
Supplement: Additional file 3: Table S3. — Permutational multivariate analysis of variance (PERMANOVA) tests of the bacterial and fungal gut microbiota on the unweighted and weighted UniFrac distances and the Bray-Curtis dissimilarity according to the severity of the autistic phenotype. (PDF 11 kb) [file 40168_2017_242_MOESM3_ESM.pdf]

Supplementary Table 3: Permutational multivariate analysis of variance (PERMANOVA) tests of the bacterial and fungal gut microbiota on the unweighted and weighted UniFrac distances and the Bray-Curtis dissimilarity according to the severity of the autistic phenotype.

**Bacterial microbiota**	**Metric**	**F**	R^2	***p-value***
**AD severe** (n=36) *vs* **AD moderate** (n=4)	Unweighted Unifrac	1.04	0.02	0.43
	Weighted Unifrac	1.19	0.03	0.27
	Bray-Curtis	1.27	0.03	0.24

**Fungal microbiota**	**Metric**	**F**	R^2	***p-value***
**AD severe** (n=31) *vs* **AD moderate** (n=4)	Unweighted Unifrac	0.44	0.01	0.92
	Weighted Unifrac	0.77	0.02	0.52
	Bray-Curtis	0.54	0.01	0.69
