# Supplementary figures and images for: New evidences on the altered gut microbiota in autism spectrum disorders
Source: Microbiome. 2017 Feb 22;5:24. doi: 10.1186/s40168-017-0242-1 (PMC5320696; doi:10.1186/s40168-017-0242-1)

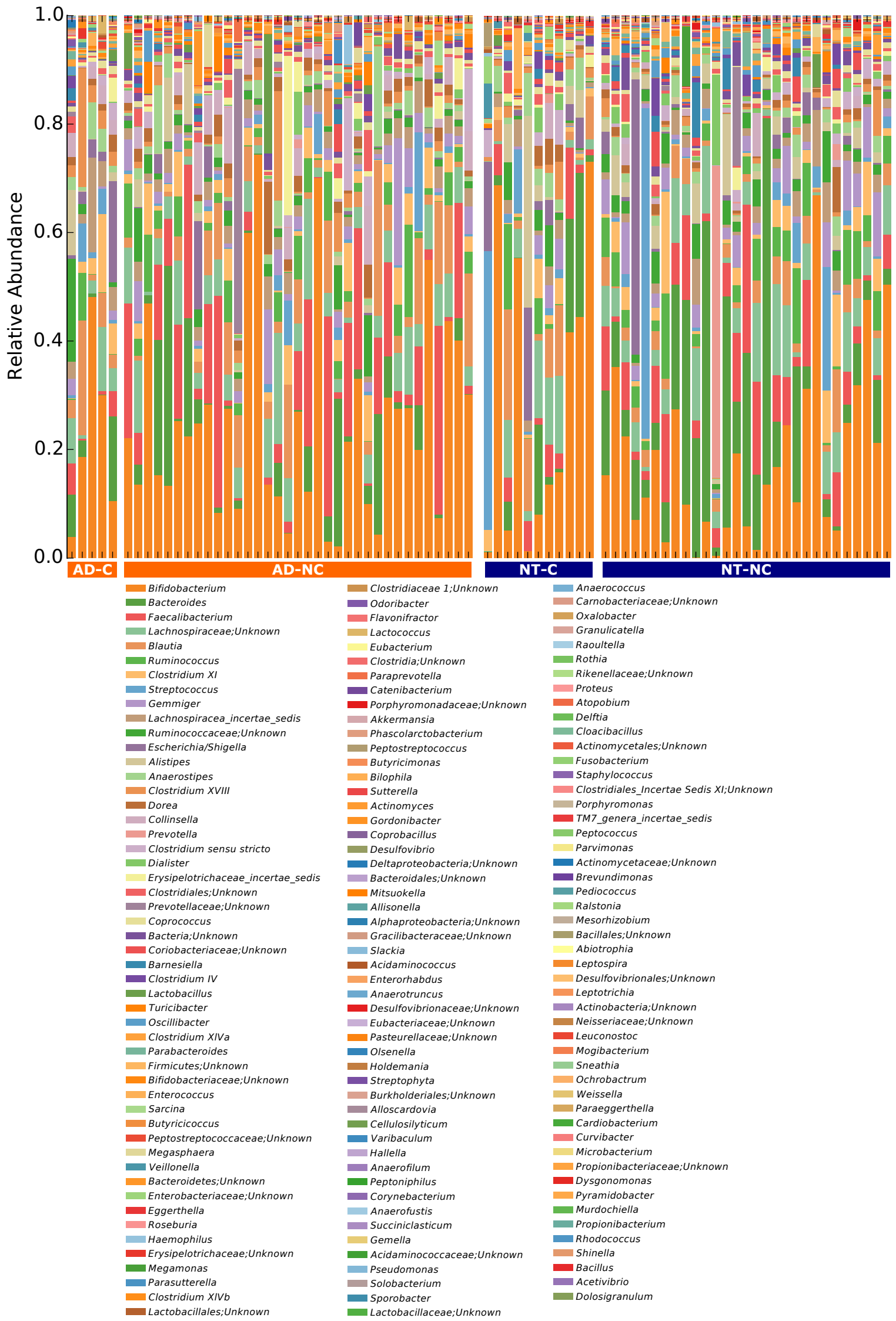

Supplement: Additional file 4: Figure S1. — Relative abundances at the genus level of the bacterial gut microbiota of autistic (AD) and neurotypical (NT) subjects both constipated (C) and non-constipated (NC). (PDF 1830 kb) [file 40168_2017_242_MOESM4_ESM.pdf]

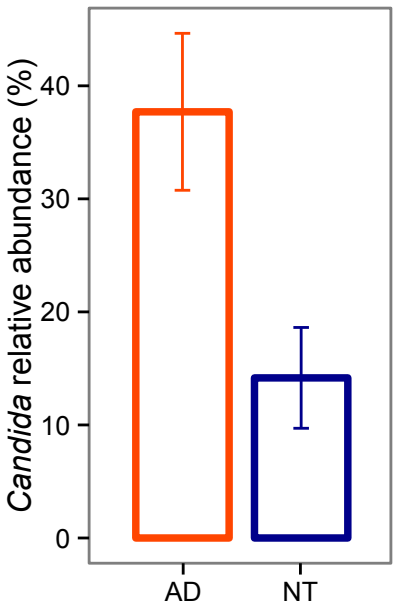

Supplement: Additional file 10: Figure S3. — Candida relative abundance in autistic (AD) and neurotypical (NT) subjects. Candida relative abundances are reported as mean ± standard error. (PDF 19 kb) [file 40168_2017_242_MOESM10_ESM.pdf]
