# Supplementary material for: New evidences on the altered gut microbiota in autism spectrum disorders
Source: Microbiome. 2017 Feb 22;5:24. doi: 10.1186/s40168-017-0242-1 (PMC5320696; doi:10.1186/s40168-017-0242-1)
Supplement: Additional file 5: Table S4. — Mean relative abundance (%) ± standard deviation (SD) of bacterial taxa at genus levels in autistic (AD) subjects and neurotypical (NT) controls subjects both constipated (C) and non-constipated (NC). (PDF 303 kb) [file 40168_2017_242_MOESM5_ESM.pdf]

Supplementary Table 4: Mean relative abundance (%) ± standard deviation (SD) of bacterial taxa at genus levels in autistic (AD) and neurotypical (NT) subjects both constipated (C) and non-constipated (NC).

	AD		NT		AD-NC		AD-C		NT-NC		NT-C	
Genus	**mean**	**SD**	**mean**	**SD**	**mean**	**SD**	**mean**	**SD**	**mean**	**SD**	**mean**	**SD**
Bifidobacterium	24.9000	18.1659	21.7741	20.2050	26.2459	19.3322	22.2564	17.4641	18.7076	16.1969	29.8585	27.5398
Bacteroides	5.6545	9.2705	12.6658	15.8310	4.8177	9.0817	5.4283	6.4654	14.8957	17.2179	6.7872	9.7427
Faecalibacterium	10.2981	11.7366	6.2955	6.1588	11.5902	11.9463	2.2778	2.7370	7.2501	6.6415	3.7789	3.8489
Lachnospiraceae;Unknown	6.3966	4.5323	6.4080	4.8425	6.8836	4.5400	5.4699	4.5756	6.6496	4.4803	5.7713	5.8849
Blautia	6.2533	6.7229	4.0792	4.3493	6.8504	6.8236	6.9322	8.1733	3.1402	2.5915	6.5549	6.7580
Ruminococcus	3.4483	3.9080	3.9006	4.4747	3.8566	4.0712	1.7964	3.8777	4.5480	4.2181	2.1940	4.8837
Clostridium XI	3.5446	3.2881	3.0968	3.9781	3.3952	3.0237	5.0716	5.6175	3.4110	4.4716	2.2685	2.1657
Streptococcus	1.7234	3.3565	4.6497	12.6877	1.7628	3.6635	2.8009	2.9063	4.0161	11.7806	6.3204	15.3292
Gemmiger	3.0106	4.2047	3.0436	3.3370	3.5163	4.6064	1.0224	1.3213	3.7067	3.4961	1.2953	2.1374
Lachnospiracea_incertae_sedis	3.0411	2.7147	2.4181	2.2914	2.7028	1.3934	6.2676	6.3233	2.5923	2.5157	1.9589	1.5609
Ruminococcaceae;Unknown	2.3921	3.6106	2.4617	2.3196	1.6564	1.9501	4.3666	8.1926	2.6479	2.1183	1.9708	2.8379
Escherichia/Shigella	1.4706	3.8944	3.1831	8.7676	0.4731	1.2099	5.3189	7.4712	2.5425	9.3382	4.8721	7.1628
Alistipes	1.2848	3.1149	3.1659	5.9674	0.6116	2.1424	2.8081	5.2410	2.4785	3.0930	4.9780	10.3494
Anaerostipes	2.4452	3.4284	1.9146	1.8001	2.6335	3.7884	3.0161	2.4568	1.4996	1.2319	3.0089	2.5636
Clostridium XVIII	2.4645	4.6033	1.2291	1.6523	1.8462	3.6987	6.6457	8.5055	1.0161	1.5696	1.7908	1.8084
Dorea	2.1164	2.2171	1.1022	1.5706	2.3000	2.4804	1.4492	1.0954	1.0497	1.5281	1.2407	1.7473
Collinsella	2.4212	3.0206	0.7377	1.7124	2.2217	2.8077	2.5441	1.6025	0.7855	1.6358	0.6117	1.9798
Clostridium sensu stricto	1.7014	3.2238	1.1334	1.4337	1.9206	3.6399	1.2483	1.2948	1.0142	1.0385	1.4477	2.2014
Erysipelotrichaceae_incertae_sedis	1.6220	4.7726	0.4596	0.8299	1.7466	5.4441	1.2091	1.8392	0.4071	0.8734	0.5982	0.7218
Clostridiales;Unknown	1.0777	1.3254	0.9136	1.0758	0.9062	1.3068	1.1163	1.0965	0.9408	1.0560	0.8419	1.1760
Dialister	0.5714	1.3153	1.4077	2.7441	0.6744	1.4609	0.0464	0.0773	1.6265	3.0762	0.8311	1.5381
Prevotellaceae;Unknown	1.3324	7.7123	0.4882	2.8855	1.7257	8.9074	0.1938	0.4234	0.6721	3.3867	0.0032	0.0108
Coprococcus	0.8026	0.5336	0.8585	0.6871	0.8014	0.5555	0.9059	0.5469	0.8047	0.6772	1.0003	0.7257
Unknown	0.8041	0.8209	0.8570	1.1623	0.9125	0.8936	0.3329	0.2022	1.0150	1.3098	0.4404	0.4499
Prevotella	0.0501	0.2717	1.5614	9.0676	0.0652	0.3137	0.0000	0.0000	2.0918	10.6478	0.1632	0.4924
Coriobacteriaceae;Unknown	0.5268	0.9162	0.8099	1.3768	0.3230	0.3489	0.1153	0.1998	0.9298	1.5587	0.4939	0.6622
Lactobacillus	1.0018	3.5838	0.2511	1.0308	1.1847	4.1198	0.4446	0.9513	0.2577	1.1633	0.2340	0.5951

Clostridium IV	0.4069	0.6500	0.8271	1.3817	0.3021	0.5829	0.5243	0.9899	0.8672	1.5054	0.7214	1.0408
Turicibacter	0.7895	1.5079	0.3824	0.7666	0.9308	1.7086	0.0725	0.0489	0.4725	0.8798	0.1448	0.1982
Barnesiella	0.2219	0.6002	0.8845	1.9255	0.0535	0.1953	0.5041	0.9901	1.1470	2.1907	0.1924	0.5430
Oscillibacter	0.4162	0.9883	0.5668	0.7707	0.3132	1.0566	0.3828	0.6825	0.6461	0.8050	0.3577	0.6602
Clostridium XlVa	0.4605	0.4844	0.4947	0.6779	0.4421	0.4928	0.5552	0.4492	0.5299	0.7682	0.4020	0.3561
Firmicutes;Unknown	0.3529	0.4567	0.5375	0.7557	0.3396	0.4758	0.2306	0.1516	0.6582	0.8487	0.2194	0.2379
Parabacteroides	0.1896	0.3689	0.6876	1.1413	0.0765	0.1286	0.4530	0.6515	0.9002	1.2781	0.1270	0.1711
Bifidobacteriaceae;Unknown	0.4625	0.3040	0.3696	0.3211	0.4831	0.3105	0.4006	0.2835	0.3687	0.3218	0.3718	0.3348
Enterococcus	0.1428	0.4268	0.4562	1.8010	0.1008	0.2299	0.5445	1.0733	0.1857	0.5507	1.1694	3.3210
Sarcina	0.3079	0.7661	0.2204	0.4950	0.3380	0.8600	0.1379	0.1350	0.1847	0.3732	0.3145	0.7432
Butyricicoccus	0.3326	0.5226	0.1754	0.2275	0.3974	0.5849	0.1379	0.1575	0.1945	0.2308	0.1248	0.2209
Peptostreptococcaceae;Unknown	0.2614	0.2492	0.1574	0.1747	0.2525	0.1991	0.4363	0.4903	0.1572	0.1919	0.1578	0.1257
Megasphaera	0.3021	1.3036	0.0961	0.4098	0.3196	1.4333	0.0202	0.0419	0.1238	0.4801	0.0232	0.0382
Veillonella	0.0627	0.1681	0.3180	1.0394	0.0336	0.0701	0.2972	0.4005	0.0924	0.1538	0.9127	1.9001
Enterobacteriaceae;Unknown	0.0852	0.1755	0.2826	0.8837	0.0375	0.0637	0.4113	0.3301	0.0781	0.1533	0.8219	1.5934
Eggerthella	0.1005	0.1384	0.2366	0.2953	0.0912	0.1315	0.1890	0.1960	0.1781	0.2389	0.3907	0.3800
Bacteroidetes;Unknown	0.2511	1.5845	0.0639	0.3946	0.0004	0.0022	0.0000	0.0000	0.0881	0.4633	0.0000	0.0000
Haemophilus	0.2372	1.1607	0.0727	0.1800	0.3259	1.3354	0.0048	0.0106	0.0676	0.1746	0.0859	0.2017
Roseburia	0.1624	0.1926	0.1434	0.1685	0.1970	0.2090	0.0690	0.0729	0.1640	0.1818	0.0892	0.1175
Erysipelotrichaceae;Unknown	0.1806	0.2920	0.0993	0.1429	0.1660	0.2107	0.3864	0.6496	0.0818	0.0820	0.1454	0.2399
Megamonas	0.0000	0.0000	0.2794	1.7487	0.0000	0.0000	0.0000	0.0000	0.3854	2.0536	0.0000	0.0000
Parasutterella	0.1560	0.6878	0.0898	0.1907	0.0232	0.0775	0.0321	0.0623	0.1084	0.2089	0.0405	0.1266
Lactobacillales;Unknown	0.1061	0.1861	0.1259	0.2441	0.1068	0.1882	0.2128	0.2320	0.0990	0.1875	0.1967	0.3554
Clostridiaceae 1;Unknown	0.1388	0.2252	0.0877	0.1618	0.1523	0.2481	0.1415	0.1486	0.0615	0.0766	0.1567	0.2800
Clostridium XlVb	0.1277	0.3296	0.0901	0.2042	0.1201	0.3331	0.2627	0.4710	0.0717	0.1485	0.1383	0.3120
Odoribacter	0.0878	0.2304	0.1266	0.1962	0.0264	0.0839	0.2794	0.5542	0.1658	0.2159	0.0232	0.0588
Flavonifractor	0.0740	0.1251	0.1367	0.1392	0.0510	0.0818	0.0820	0.0971	0.1261	0.1407	0.1648	0.1376
Lactococcus	0.1953	0.6485	0.0140	0.0338	0.1287	0.4673	0.8156	1.3953	0.0100	0.0320	0.0243	0.0378
Eubacterium	0.0939	0.1480	0.0942	0.1122	0.0691	0.1275	0.0832	0.1264	0.1082	0.1164	0.0573	0.0955
Clostridia;Unknown	0.0892	0.1915	0.0811	0.1913	0.0533	0.1709	0.0713	0.1466	0.0570	0.1236	0.1448	0.3061
Catenibacterium	0.1268	0.6975	0.0198	0.1240	0.0240	0.1258	0.0000	0.0000	0.0273	0.1457	0.0000	0.0000
Paraprevotella	0.1097	0.6417	0.0366	0.1304	0.1416	0.7405	0.0464	0.1037	0.0502	0.1516	0.0005	0.0018

Akkermansia	0.0380	0.0833	0.1031	0.1674	0.0277	0.0748	0.0380	0.0547	0.0816	0.1679	0.1600	0.1597
Phascolarctobacterium	0.0724	0.2345	0.0525	0.1542	0.0092	0.0241	0.1201	0.2138	0.0717	0.1781	0.0016	0.0054
Porphyromonadaceae;Unknown	0.0196	0.1054	0.1015	0.3790	0.0020	0.0078	0.0119	0.0266	0.1386	0.4415	0.0038	0.0107
Peptostreptococcus	0.0004	0.0021	0.1089	0.6841	0.0004	0.0022	0.0012	0.0027	0.0006	0.0033	0.3945	1.3044
Butyricimonas	0.0259	0.0718	0.0367	0.1108	0.0018	0.0063	0.1153	0.1596	0.0504	0.1280	0.0005	0.0018
Bilophila	0.0108	0.0247	0.0477	0.1039	0.0039	0.0101	0.0095	0.0108	0.0640	0.1184	0.0049	0.0074
Actinomyces	0.0260	0.0252	0.0294	0.0404	0.0258	0.0200	0.0262	0.0486	0.0219	0.0254	0.0492	0.0629
Sutterella	0.0022	0.0084	0.0508	0.1827	0.0016	0.0062	0.0083	0.0186	0.0701	0.2123	0.0000	0.0000
Gordonibacter	0.0199	0.0295	0.0305	0.0690	0.0164	0.0183	0.0297	0.0371	0.0305	0.0781	0.0303	0.0387
Coprobacillus	0.0104	0.0285	0.0397	0.1089	0.0055	0.0183	0.0440	0.0611	0.0375	0.1016	0.0454	0.1314
Deltaproteobacteria;Unknown	0.0486	0.2397	0.0006	0.0029	0.0000	0.0000	0.2889	0.6460	0.0002	0.0011	0.0016	0.0054
Desulfovibrio	0.0205	0.0637	0.0260	0.0609	0.0006	0.0024	0.1011	0.1392	0.0246	0.0559	0.0297	0.0752
Bacteroidales;Unknown	0.0123	0.0375	0.0278	0.0465	0.0053	0.0131	0.0499	0.0989	0.0367	0.0519	0.0043	0.0080
Mitsuokella	0.0385	0.1761	0.0000	0.0000	0.0336	0.1779	0.0000	0.0000	0.0000	0.0000	0.0000	0.0000
Allisonella	0.0324	0.1082	0.0042	0.0194	0.0131	0.0557	0.0464	0.1037	0.0057	0.0227	0.0000	0.0000
Gracilibacteraceae;Unknown	0.0250	0.0837	0.0089	0.0409	0.0010	0.0044	0.0440	0.0984	0.0123	0.0478	0.0000	0.0000
Acidaminococcus	0.0091	0.0333	0.0232	0.1286	0.0057	0.0230	0.0000	0.0000	0.0281	0.1501	0.0103	0.0341
Slackia	0.0265	0.1181	0.0052	0.0204	0.0115	0.0607	0.0095	0.0213	0.0072	0.0238	0.0000	0.0000
Anaerotruncus	0.0111	0.0272	0.0146	0.0306	0.0025	0.0057	0.0428	0.0638	0.0139	0.0334	0.0162	0.0229
Eubacteriaceae;Unknown	0.0177	0.0812	0.0071	0.0282	0.0055	0.0211	0.0036	0.0080	0.0033	0.0127	0.0173	0.0500
Alphaproteobacteria;Unknown	0.0059	0.0229	0.0175	0.0635	0.0027	0.0141	0.0000	0.0000	0.0240	0.0738	0.0005	0.0018
Pasteurellaceae;Unknown	0.0031	0.0188	0.0181	0.0840	0.0043	0.0217	0.0000	0.0000	0.0068	0.0221	0.0481	0.1575
Olsenella	0.0166	0.0677	0.0025	0.0099	0.0014	0.0049	0.0273	0.0611	0.0035	0.0115	0.0000	0.0000
Desulfovibrionaceae;Unknown	0.0120	0.0618	0.0064	0.0329	0.0133	0.0705	0.0143	0.0319	0.0084	0.0386	0.0011	0.0024
Holdemania	0.0076	0.0167	0.0088	0.0138	0.0039	0.0077	0.0071	0.0106	0.0107	0.0149	0.0038	0.0089
Streptophyta	0.0103	0.0247	0.0052	0.0194	0.0096	0.0217	0.0226	0.0473	0.0031	0.0134	0.0108	0.0304
Burkholderiales;Unknown	0.0046	0.0264	0.0098	0.0311	0.0057	0.0304	0.0036	0.0080	0.0129	0.0360	0.0016	0.0054
Enterorhabdus	0.0065	0.0256	0.0049	0.0236	0.0029	0.0152	0.0000	0.0000	0.0068	0.0276	0.0000	0.0000
Alloscardovia	0.0007	0.0028	0.0097	0.0611	0.0010	0.0031	0.0000	0.0000	0.0000	0.0000	0.0351	0.1165
Cellulosilyticum	0.0091	0.0573	0.0000	0.0000	0.0000	0.0000	0.0000	0.0000	0.0000	0.0000	0.0000	0.0000
Varibaculum	0.0006	0.0038	0.0079	0.0379	0.0000	0.0000	0.0000	0.0000	0.0094	0.0444	0.0038	0.0067
Hallella	0.0076	0.0479	0.0000	0.0000	0.0105	0.0553	0.0000	0.0000	0.0000	0.0000	0.0000	0.0000

Anaerofustis	0.0042	0.0085	0.0030	0.0084	0.0043	0.0084	0.0024	0.0033	0.0029	0.0092	0.0032	0.0062
Peptoniphilus	0.0045	0.0160	0.0027	0.0075	0.0031	0.0093	0.0000	0.0000	0.0027	0.0083	0.0027	0.0049
Anaerofilum	0.0027	0.0075	0.0043	0.0103	0.0008	0.0043	0.0071	0.0160	0.0035	0.0085	0.0065	0.0144
Succiniclasticum	0.0000	0.0000	0.0070	0.0442	0.0000	0.0000	0.0000	0.0000	0.0096	0.0519	0.0000	0.0000
Corynebacterium	0.0058	0.0113	0.0010	0.0035	0.0033	0.0058	0.0143	0.0254	0.0012	0.0040	0.0005	0.0018
Gemella	0.0037	0.0089	0.0021	0.0055	0.0031	0.0067	0.0036	0.0080	0.0018	0.0055	0.0027	0.0056
Acidaminococcaceae;Unknown	0.0006	0.0038	0.0051	0.0320	0.0000	0.0000	0.0000	0.0000	0.0070	0.0375	0.0000	0.0000
Pseudomonas	0.0027	0.0150	0.0022	0.0104	0.0033	0.0174	0.0000	0.0000	0.0020	0.0110	0.0027	0.0090
Solobacterium	0.0015	0.0042	0.0030	0.0065	0.0016	0.0044	0.0000	0.0000	0.0029	0.0069	0.0032	0.0056
Lactobacillaceae;Unknown	0.0043	0.0113	0.0000	0.0000	0.0039	0.0111	0.0059	0.0133	0.0000	0.0000	0.0000	0.0000
Sporobacter	0.0006	0.0023	0.0037	0.0088	0.0000	0.0000	0.0012	0.0027	0.0039	0.0089	0.0032	0.0090
Carnobacteriaceae;Unknown	0.0000	0.0000	0.0040	0.0132	0.0000	0.0000	0.0000	0.0000	0.0025	0.0093	0.0081	0.0203
Anaerococcus	0.0009	0.0039	0.0031	0.0126	0.0010	0.0044	0.0000	0.0000	0.0010	0.0039	0.0086	0.0231
Granulicatella	0.0004	0.0021	0.0030	0.0143	0.0006	0.0024	0.0000	0.0000	0.0008	0.0035	0.0086	0.0268
Oxalobacter	0.0019	0.0078	0.0015	0.0077	0.0002	0.0011	0.0036	0.0053	0.0020	0.0090	0.0000	0.0000
Raoultella	0.0012	0.0059	0.0022	0.0066	0.0004	0.0022	0.0071	0.0160	0.0006	0.0033	0.0065	0.0105
Rothia	0.0021	0.0061	0.0012	0.0043	0.0025	0.0067	0.0024	0.0053	0.0006	0.0024	0.0027	0.0072
Rikenellaceae;Unknown	0.0012	0.0051	0.0019	0.0074	0.0000	0.0000	0.0071	0.0129	0.0027	0.0086	0.0000	0.0000
Proteus	0.0013	0.0068	0.0016	0.0086	0.0000	0.0000	0.0083	0.0186	0.0000	0.0000	0.0059	0.0162
Delftia	0.0027	0.0169	0.0000	0.0000	0.0037	0.0195	0.0000	0.0000	0.0000	0.0000	0.0000	0.0000
Atopobium	0.0004	0.0016	0.0022	0.0093	0.0006	0.0018	0.0000	0.0000	0.0012	0.0066	0.0049	0.0143
Actinomycetales;Unknown	0.0016	0.0050	0.0007	0.0028	0.0020	0.0057	0.0012	0.0027	0.0008	0.0031	0.0005	0.0018
Cloacibacillus	0.0015	0.0094	0.0009	0.0042	0.0000	0.0000	0.0000	0.0000	0.0008	0.0044	0.0011	0.0036
Fusobacterium	0.0001	0.0009	0.0021	0.0096	0.0002	0.0011	0.0000	0.0000	0.0010	0.0055	0.0049	0.0161
Staphylococcus	0.0012	0.0034	0.0006	0.0023	0.0014	0.0037	0.0000	0.0000	0.0006	0.0024	0.0005	0.0018
Clostridiales_Incertae Sedis XI;Unknown	0.0018	0.0095	0.0000	0.0000	0.0004	0.0022	0.0000	0.0000	0.0000	0.0000	0.0000	0.0000
Porphyromonas	0.0012	0.0051	0.0004	0.0021	0.0006	0.0024	0.0000	0.0000	0.0004	0.0022	0.0005	0.0018
TM7_genera_incertae_sedis	0.0012	0.0066	0.0004	0.0021	0.0014	0.0076	0.0000	0.0000	0.0004	0.0022	0.0005	0.0018
Peptococcus	0.0001	0.0009	0.0013	0.0085	0.0000	0.0000	0.0000	0.0000	0.0018	0.0099	0.0000	0.0000
Parvimonas	0.0007	0.0024	0.0007	0.0031	0.0008	0.0026	0.0000	0.0000	0.0006	0.0033	0.0011	0.0024
Actinomycetaceae;Unknown	0.0006	0.0029	0.0007	0.0039	0.0008	0.0034	0.0000	0.0000	0.0010	0.0045	0.0000	0.0000
Brevundimonas	0.0010	0.0066	0.0003	0.0019	0.0014	0.0076	0.0000	0.0000	0.0004	0.0022	0.0000	0.0000

Ralstonia	0.0012	0.0052	0.0000	0.0000	0.0016	0.0060	0.0000	0.0000	0.0000	0.0000	0.0000	0.0000
Mesorhizobium	0.0009	0.0034	0.0001	0.0009	0.0012	0.0039	0.0000	0.0000	0.0000	0.0000	0.0005	0.0018
Bacillales;Unknown	0.0009	0.0056	0.0000	0.0000	0.0012	0.0065	0.0000	0.0000	0.0000	0.0000	0.0000	0.0000
Abiotrophia	0.0003	0.0019	0.0006	0.0023	0.0004	0.0022	0.0000	0.0000	0.0004	0.0015	0.0011	0.0036
Leptospira	0.0009	0.0056	0.0000	0.0000	0.0012	0.0065	0.0000	0.0000	0.0000	0.0000	0.0000	0.0000
Actinobacteria;Unknown	0.0003	0.0019	0.0004	0.0028	0.0000	0.0000	0.0000	0.0000	0.0000	0.0000	0.0016	0.0054
Leptotrichia	0.0000	0.0000	0.0007	0.0047	0.0000	0.0000	0.0000	0.0000	0.0010	0.0055	0.0000	0.0000
Neisseriaceae;Unknown	0.0000	0.0000	0.0007	0.0047	0.0000	0.0000	0.0000	0.0000	0.0000	0.0000	0.0027	0.0090
Desulfovibrionales;Unknown	0.0001	0.0009	0.0006	0.0038	0.0000	0.0000	0.0012	0.0027	0.0000	0.0000	0.0022	0.0072
Leuconostoc	0.0001	0.0009	0.0004	0.0021	0.0002	0.0011	0.0000	0.0000	0.0004	0.0022	0.0005	0.0018
Paraeggerthella	0.0000	0.0000	0.0004	0.0028	0.0000	0.0000	0.0000	0.0000	0.0006	0.0033	0.0000	0.0000
Weissella	0.0000	0.0000	0.0004	0.0021	0.0000	0.0000	0.0000	0.0000	0.0006	0.0024	0.0000	0.0000
Mogibacterium	0.0000	0.0000	0.0004	0.0028	0.0000	0.0000	0.0000	0.0000	0.0006	0.0033	0.0000	0.0000
Sneathia	0.0004	0.0028	0.0000	0.0000	0.0006	0.0033	0.0000	0.0000	0.0000	0.0000	0.0000	0.0000
Ochrobactrum	0.0004	0.0028	0.0000	0.0000	0.0000	0.0000	0.0000	0.0000	0.0000	0.0000	0.0000	0.0000
Cardiobacterium	0.0000	0.0000	0.0004	0.0028	0.0000	0.0000	0.0000	0.0000	0.0006	0.0033	0.0000	0.0000
Microbacterium	0.0000	0.0000	0.0003	0.0013	0.0000	0.0000	0.0000	0.0000	0.0000	0.0000	0.0011	0.0024
Propionibacteriaceae;Unknown	0.0000	0.0000	0.0003	0.0019	0.0000	0.0000	0.0000	0.0000	0.0004	0.0022	0.0000	0.0000
Dysgonomonas	0.0001	0.0009	0.0001	0.0009	0.0000	0.0000	0.0000	0.0000	0.0002	0.0011	0.0000	0.0000
Pediococcus	0.0000	0.0000	0.0003	0.0019	0.0000	0.0000	0.0000	0.0000	0.0004	0.0022	0.0000	0.0000
Murdochiella	0.0001	0.0009	0.0001	0.0009	0.0002	0.0011	0.0000	0.0000	0.0000	0.0000	0.0005	0.0018
Curvibacter	0.0003	0.0019	0.0000	0.0000	0.0004	0.0022	0.0000	0.0000	0.0000	0.0000	0.0000	0.0000
Pyramidobacter	0.0003	0.0019	0.0000	0.0000	0.0000	0.0000	0.0000	0.0000	0.0000	0.0000	0.0000	0.0000
Rhodococcus	0.0001	0.0009	0.0000	0.0000	0.0002	0.0011	0.0000	0.0000	0.0000	0.0000	0.0000	0.0000
Propionibacterium	0.0001	0.0009	0.0000	0.0000	0.0002	0.0011	0.0000	0.0000	0.0000	0.0000	0.0000	0.0000
Bacillus	0.0000	0.0000	0.0001	0.0009	0.0000	0.0000	0.0000	0.0000	0.0000	0.0000	0.0005	0.0018
Dolosigranulum	0.0000	0.0000	0.0001	0.0009	0.0000	0.0000	0.0000	0.0000	0.0000	0.0000	0.0005	0.0018
Acetivibrio	0.0000	0.0000	0.0001	0.0009	0.0000	0.0000	0.0000	0.0000	0.0000	0.0000	0.0005	0.0018
Shinella	0.0000	0.0000	0.0001	0.0009	0.0000	0.0000	0.0000	0.0000	0.0002	0.0011	0.0000	0.0000
