# Supplementary material for: New evidences on the altered gut microbiota in autism spectrum disorders
Source: Microbiome. 2017 Feb 22;5:24. doi: 10.1186/s40168-017-0242-1 (PMC5320696; doi:10.1186/s40168-017-0242-1)
Supplement: Additional file 6: — Spearman’s correlation analysis among the most abundant bacterial genera and the constipation status of the subjects of the study cohort. (PDF 154 kb) [file 40168_2017_242_MOESM6_ESM.pdf]

Supplementary Table 5: Spearman's correlation analysis among the most abundant bacterial genera and the constipation status of the subjects of the study cohort.

Genus	**Spearman's *r***	***p*-values**	**FDR-corrected *p***
Gemmiger	-0.3950	0.0005	0.0097
Clostridium XVIII	0.3811	0.0008	0.0097
Ruminococcus	-0.3606	0.0016	0.0128
Escherichia/Shigella	0.3061	0.0080	0.0480
Faecalibacterium	-0.2843	0.0141	0.0676
Dialister	-0.2258	0.0531	0.2123
Anaerostipes	0.1952	0.0956	0.2550
Erysipelotrichaceae incertae sedis	0.1953	0.0955	0.2550
Turicibacter	-0.1960	0.0941	0.2550
Collinsella	-0.1654	0.1590	0.3816
Oscillibacter	-0.1440	0.2210	0.4822
Dorea	-0.1360	0.2479	0.4958
Streptococcus	0.1245	0.2906	0.5083
Lactobacillus	0.1230	0.2965	0.5083
Alistipes	0.1079	0.3603	0.5686
Coprococcus	0.1037	0.3791	0.5686
Bacteroides	-0.0922	0.4346	0.6135
Blautia	0.0645	0.5848	0.7387
Clostridium IV	-0.0692	0.5581	0.7387
Clostridium XlVa	0.0553	0.6396	0.7675
Bifidobacterium	0.0430	0.7158	0.8181
Lachnospiracea incertae sedis	0.0300	0.7999	0.8726
Clostridium sensu stricto	0.0108	0.9275	0.9678
Clostridium XI	0.0008	0.9948	0.9948
