# Supplementary material for: New evidences on the altered gut microbiota in autism spectrum disorders
Source: Microbiome. 2017 Feb 22;5:24. doi: 10.1186/s40168-017-0242-1 (PMC5320696; doi:10.1186/s40168-017-0242-1)
Supplement: Additional file 7: Table S6. — Permutational multivariate analysis of variance (PERMANOVA) tests of the fungal gut microbiota on the unweighted and weighted UniFrac distances and the Bray-Curtis dissimilarity according to individuals’ health status and constipation. (PDF 159 kb) [file 40168_2017_242_MOESM7_ESM.pdf]

Supplementary Table 6: Permutational multivariate analysis of variance (PERMANOVA) tests of the fungal gut microbiota on the unweighted and weighted UniFrac distances and the Bray-Curtis dissimilarity according to individuals' health status and constipation.

	**Metric**	**F**	**R^2**	***p-value***
**NT** (n=38) *vs* **AD** (n=35)	Unweighted Unifrac	1.55	0.02	0.095
	Weighted Unifrac	2.59	0.02	0.040
	Bray-Curtis	2.91	0.03	0.029
**AD-C** (n=5) *vs* **AD-NC** (n=24)	Unweighted Unifrac	0.61	0.02	0.777
	Weighted Unifrac	0.63	0.02	0.588
	Bray-Curtis	0.57	0.02	0.623
**NT-C** (n=11) *vs* **NT-NC** (n=27)	Unweighted Unifrac	1.06	0.02	0.336
	Weighted Unifrac	1.26	0.03	0.240
	Bray-Curtis	2.36	0.06	0.046
**AD-C** (n=5) *vs* **NT-C** (n=11)	Unweighted Unifrac	0.82	0.05	0.570
	Weighted Unifrac	1.60	0.10	0.169
	Bray-Curtis	1.48	0.09	0.204
**AD-NC** (n=24) *vs* **NT-NC** (n=27)	Unweighted Unifrac	1.27	0.02	0.199
	Weighted Unifrac	2.47	0.04	0.049
	Bray-Curtis	3.01	0.05	0.020

NT, neurotypical subjects; AD, autistic subjects; NT-C, constipated neurotypical subjects; NT-NC, non-constipated neurotypical subjects; AD-C, constipated autistic subjects; AD-NC, non-constipated autistic subjects.
