# Supplementary material for: New evidences on the altered gut microbiota in autism spectrum disorders
Source: Microbiome. 2017 Feb 22;5:24. doi: 10.1186/s40168-017-0242-1 (PMC5320696; doi:10.1186/s40168-017-0242-1)
Supplement: Additional file 8: Figure S2. — Relative abundances at the genus level of the fungal gut microbiota of autistic (AD) and neurotypical (NT) subjects both constipated (C) and non-constipated (NC). (PDF 346 kb) [file 40168_2017_242_MOESM8_ESM.pdf]

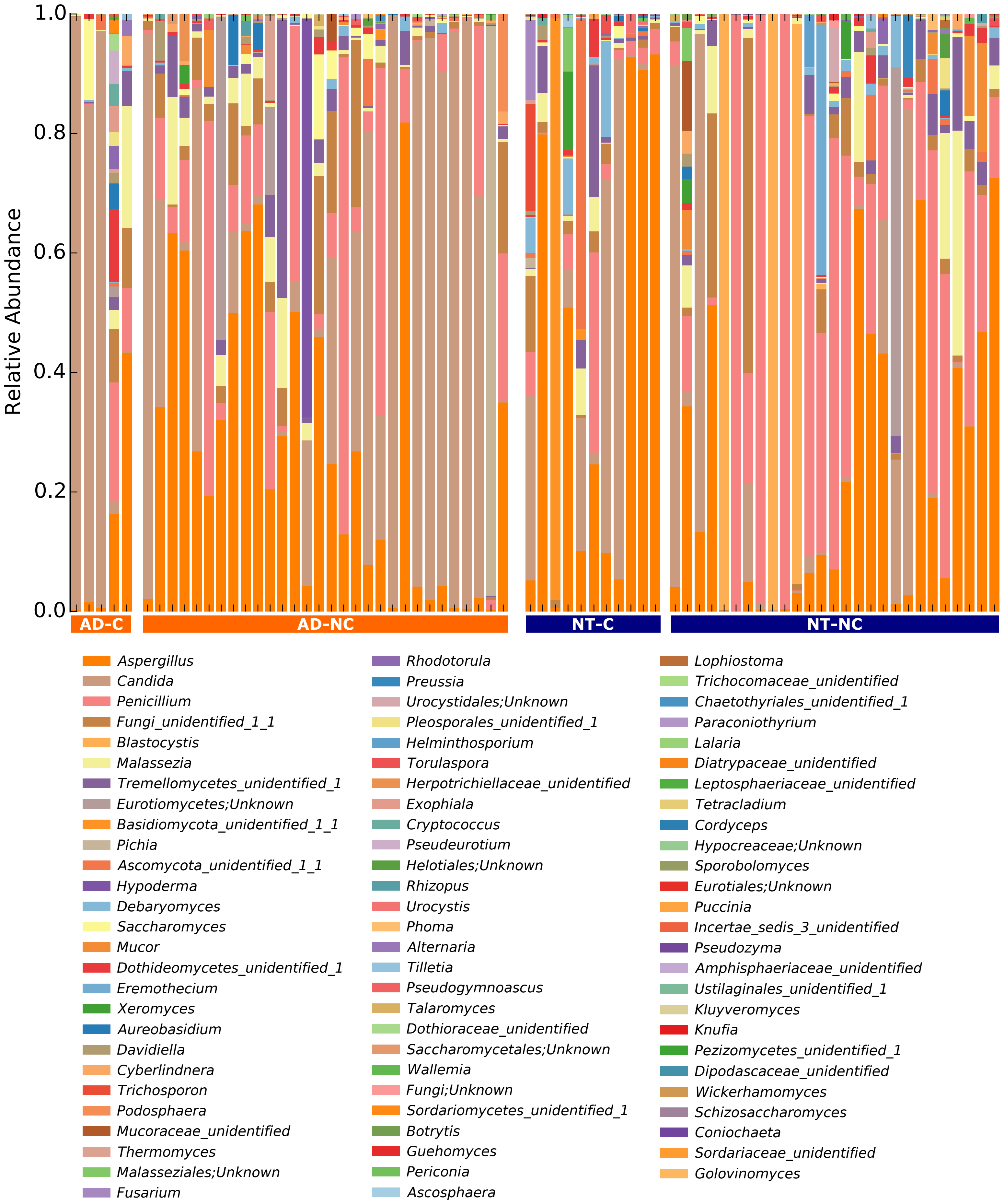
Relative Abundance
1.0
0.8
0.6
0.4
0.2
0.0
AD-C
AD-NC
NT-C
NT-NC
Aspergillus
Candida
Penicillium
Fungi_unidentified_1_1
Blastocystis
Malassezia
Tremellomycetes_unidentified_1
Eurotiomycetes;Unknown
Basidiomycota_unidentified_1_1
Pichia
Ascomycota_unidentified_1_1
Hypoderma
Debaryomyces
Saccharomyces
Mucor
Dothideomycetes_unidentified_1
Eremothecium
Xeromyces
Aureobasidium
Davidiella
Cyberlindnera
Trichosporon
Podosphaera
Mucoraceae_unidentified
Thermomyces
Malasseziales;Unknown
Fusarium
Rhodotorula
Preussia
Urocystidales;Unknown
Pleosporales_unidentified_1
Helminthosporium
Torulaspora
Herpotrichiellaceae_unidentified
Exophiala
Cryptococcus
Pseudeurotium
Helotiales;Unknown
Rhizopus
Urocystis
Phoma
Alternaria
Tilletia
Pseudogymnoascus
Talaromyces
Dothioraceae_unidentified
Saccharomycetales;Unknown
Wallemia
Fungi;Unknown
Sordariomycetes_unidentified_1
Botrytis
Guehomyces
Periconia
Ascosphaera
Lophiostoma
Trichocomaceae_unidentified
Chaetothyriales_unidentified_1
Paraconiothyrium
Lalaria
Diatrypaceae_unidentified
Leptosphaeriaceae_unidentified
Tetracladium
Cordyceps
Hypocreaceae;Unknown
Sporobolomyces
Eurotiales;Unknown
Puccinia
Incertae_sedis_3_unidentified
Pseudozyma
Amphisphaeriaceae_unidentified
Ustilaginales_unidentified_1
Kluyveromyces
Knufia
Pezizomycetes_unidentified_1
Dipodascaceae_unidentified
Wickerhamomyces
Schizosaccharomyces
Coniochaeta
Sordariaceae_unidentified
Golovinomyces
