# Supplementary material for: New evidences on the altered gut microbiota in autism spectrum disorders
Source: Microbiome. 2017 Feb 22;5:24. doi: 10.1186/s40168-017-0242-1 (PMC5320696; doi:10.1186/s40168-017-0242-1)
Supplement: Additional file 9: Table S7. — Mean relative abundance (%) ± standard deviation (SD) of fungal taxa at genus levels in autistic (AD) subjects and neurotypical (NT) controls subjects both constipated (C) and non-constipated (NC). (PDF 199 kb) [file 40168_2017_242_MOESM9_ESM.pdf]

Supplementary Table 7: Mean relative abundance (%) ± standard deviation (SD) of fungal taxa at genus levels in autistic (AD) and neurotypical (NT) subjects both constipated (C) and non-constipated (NC).

	**AD**		**NT**		**AD-NC**		**AD-C**		**NT-NC**		**NT-C**	
Genus	**mean**	**SD**	**mean**	**SD**	**mean**	**SD**	**mean**	**SD**	**mean**	**SD**	**mean**	**SD**
Aspergillus	24.225	23.867	28.029	30.298	23.468	23.512	12.378	18.558	22.299	24.064	42.094	39.808
Candida	37.701	41.069	14.164	27.493	37.431	40.429	56.324	50.747	12.053	26.803	19.343	29.788
Penicillium	13.241	19.683	23.482	30.851	15.399	22.740	6.127	8.946	30.810	33.553	5.494	9.671
Fungi_unidentified_1_1	4.887	7.175	4.350	10.143	5.075	8.112	3.863	5.164	5.081	11.816	2.558	3.569
Blastocystis	0.000	0.000	7.742	26.718	0.000	0.000	0.000	0.000	10.896	31.308	0.000	0.000
Malassezia	3.047	5.128	3.293	7.523	2.661	4.739	4.818	8.844	3.827	8.750	1.982	2.769
Tremellomycetes_unidentified_1	3.103	7.905	2.998	4.488	3.405	9.445	1.620	2.523	2.811	3.411	3.455	6.631
Eurotiomycetes;Unknown	2.038	9.419	1.634	9.987	0.017	0.085	0.353	0.789	2.296	11.848	0.009	0.031
Pichia	2.762	16.126	0.044	0.270	4.028	19.470	0.000	0.000	0.000	0.000	0.151	0.501
Basidiomycota_unidentified_1_1	0.050	0.298	2.618	15.795	0.074	0.360	0.000	0.000	0.008	0.028	9.025	29.317
Ascomycota_unidentified_1_1	0.365	1.381	2.011	8.528	0.402	1.603	0.602	1.080	0.796	2.327	4.994	15.533
Hypoderma	1.917	11.285	0.000	0.000	2.795	13.627	0.000	0.000	0.000	0.000	0.000	0.000
Debaryomyces	0.288	0.622	1.418	3.261	0.260	0.595	0.208	0.220	0.769	1.690	3.011	5.290
Saccharomyces	1.270	3.333	0.273	0.437	1.246	3.032	2.721	6.084	0.265	0.378	0.293	0.578
Mucor	0.344	1.623	1.123	3.739	0.498	1.953	0.021	0.046	1.558	4.383	0.057	0.097
Dothideomycetes_unidentified_1	0.688	2.088	0.697	1.304	0.394	0.652	2.679	5.363	0.635	1.047	0.850	1.845
Eremothecium	0.000	0.000	1.112	6.804	0.000	0.000	0.000	0.000	1.561	8.072	0.009	0.031
Xeromyces	0.095	0.544	0.618	2.401	0.138	0.657	0.000	0.000	0.385	1.421	1.189	3.945
Aureobasidium	0.504	1.722	0.199	0.767	0.199	0.889	0.852	1.904	0.235	0.898	0.113	0.269
Davidiella	0.368	1.166	0.246	0.567	0.169	0.358	0.395	0.768	0.188	0.476	0.387	0.755
Cyberlindnera	0.312	0.918	0.213	0.728	0.234	0.503	0.997	2.229	0.285	0.854	0.038	0.125
Trichosporon	0.006	0.035	0.473	2.914	0.009	0.042	0.000	0.000	0.000	0.000	1.633	5.417
Podosphaera	0.475	2.754	0.000	0.000	0.684	3.327	0.000	0.000	0.000	0.000	0.000	0.000
Mucoraceae_unidentified	0.131	0.772	0.314	1.903	0.190	0.933	0.000	0.000	0.442	2.257	0.000	0.000
Thermomyces	0.418	2.369	0.019	0.118	0.000	0.000	0.125	0.279	0.000	0.000	0.066	0.219
Malasseziales;Unknown	0.027	0.158	0.336	1.456	0.039	0.191	0.000	0.000	0.208	1.079	0.651	2.160

Fusarium	0.003	0.018	0.353	2.156	0.000	0.000	0.000	0.000	0.000	0.000	1.218	4.005
Rhodotorula	0.148	0.663	0.158	0.692	0.056	0.196	0.768	1.718	0.188	0.818	0.085	0.160
Preussia	0.000	0.000	0.279	1.718	0.000	0.000	0.000	0.000	0.392	2.038	0.000	0.000
Urocystidales;Unknown	0.000	0.000	0.246	1.448	0.000	0.000	0.000	0.000	0.346	1.717	0.000	0.000
Pleosporales_unidentified_1	0.083	0.411	0.156	0.827	0.022	0.106	0.478	1.068	0.188	0.979	0.076	0.174
Helminthosporium	0.000	0.000	0.230	1.415	0.000	0.000	0.000	0.000	0.323	1.679	0.000	0.000
Herpotrichiellaceae_unidentified	0.228	1.334	0.000	0.000	0.333	1.610	0.000	0.000	0.000	0.000	0.000	0.000
Torulaspora	0.045	0.143	0.183	0.554	0.022	0.075	0.000	0.000	0.135	0.369	0.302	0.873
Exophiala	0.128	0.719	0.071	0.438	0.009	0.042	0.852	1.904	0.100	0.520	0.000	0.000
Cryptococcus	0.131	0.643	0.049	0.209	0.000	0.000	0.748	1.672	0.027	0.121	0.104	0.344
Pseudeurotium	0.160	0.948	0.000	0.000	0.000	0.000	1.121	2.508	0.000	0.000	0.000	0.000
Helotiales;Unknown	0.036	0.211	0.107	0.657	0.052	0.254	0.000	0.000	0.150	0.779	0.000	0.000
Rhizopus	0.074	0.439	0.044	0.253	0.108	0.530	0.000	0.000	0.058	0.300	0.009	0.031
Urocystis	0.000	0.000	0.107	0.514	0.000	0.000	0.000	0.000	0.012	0.060	0.340	0.939
Alternaria	0.083	0.457	0.022	0.135	0.009	0.042	0.540	1.207	0.031	0.160	0.000	0.000
Phoma	0.030	0.132	0.071	0.438	0.043	0.159	0.000	0.000	0.100	0.520	0.000	0.000
Tilletia	0.039	0.165	0.060	0.371	0.017	0.066	0.187	0.418	0.085	0.440	0.000	0.000
Pseudogymnoascus	0.086	0.372	0.000	0.000	0.125	0.447	0.000	0.000	0.000	0.000	0.000	0.000
Talaromyces	0.047	0.281	0.033	0.186	0.069	0.339	0.000	0.000	0.046	0.220	0.000	0.000
Dothioraceae_unidentified	0.080	0.474	0.000	0.000	0.000	0.000	0.561	1.254	0.000	0.000	0.000	0.000
Unknown	0.071	0.303	0.003	0.017	0.104	0.364	0.000	0.000	0.004	0.020	0.000	0.000
Wallemia	0.009	0.039	0.057	0.223	0.004	0.021	0.042	0.093	0.081	0.263	0.000	0.000
Sordariomycetes_unidentified_1	0.062	0.369	0.000	0.000	0.000	0.000	0.436	0.975	0.000	0.000	0.000	0.000
Fungi;Unknown	0.000	0.000	0.060	0.240	0.000	0.000	0.000	0.000	0.085	0.282	0.000	0.000
Botrytis	0.006	0.024	0.044	0.175	0.009	0.029	0.000	0.000	0.062	0.206	0.000	0.000
Guehomyces	0.000	0.000	0.046	0.194	0.000	0.000	0.000	0.000	0.042	0.200	0.057	0.188
Periconia	0.033	0.176	0.014	0.084	0.048	0.212	0.000	0.000	0.000	0.000	0.047	0.157
Ascosphaera	0.000	0.000	0.041	0.253	0.000	0.000	0.000	0.000	0.000	0.000	0.142	0.470
Lophiostoma	0.000	0.000	0.036	0.219	0.000	0.000	0.000	0.000	0.050	0.260	0.000	0.000
Trichocomaceae_unidentified	0.015	0.088	0.019	0.087	0.000	0.000	0.000	0.000	0.008	0.028	0.047	0.157
Chaetothyriales_unidentified_1	0.030	0.176	0.000	0.000	0.043	0.212	0.000	0.000	0.000	0.000	0.000	0.000
Paraconiothyrium	0.024	0.140	0.000	0.000	0.035	0.170	0.000	0.000	0.000	0.000	0.000	0.000

Lalaria	0.021	0.123	0.000	0.000	0.030	0.148	0.000	0.000	0.000	0.000	0.000	0.000
Diatrypaceae_unidentified	0.006	0.035	0.014	0.084	0.009	0.042	0.000	0.000	0.019	0.100	0.000	0.000
Leptosphaeriaceae_unidentified	0.015	0.088	0.003	0.017	0.000	0.000	0.104	0.232	0.004	0.020	0.000	0.000
Hypocreaceae;Unknown	0.003	0.018	0.011	0.040	0.004	0.021	0.000	0.000	0.004	0.020	0.028	0.067
Tetracladium	0.000	0.000	0.014	0.084	0.000	0.000	0.000	0.000	0.019	0.100	0.000	0.000
Cordyceps	0.000	0.000	0.014	0.084	0.000	0.000	0.000	0.000	0.000	0.000	0.047	0.157
Incertae_sedis_3_unidentified	0.012	0.070	0.000	0.000	0.000	0.000	0.083	0.186	0.000	0.000	0.000	0.000
Sporobolomyces	0.009	0.053	0.003	0.017	0.000	0.000	0.000	0.000	0.004	0.020	0.000	0.000
Eurotiales;Unknown	0.003	0.018	0.008	0.051	0.004	0.021	0.000	0.000	0.012	0.060	0.000	0.000
Puccinia	0.000	0.000	0.011	0.067	0.000	0.000	0.000	0.000	0.000	0.000	0.038	0.125
Amphisphaeriaceae_unidentified	0.009	0.053	0.000	0.000	0.013	0.064	0.000	0.000	0.000	0.000	0.000	0.000
Pseudozyma	0.000	0.000	0.008	0.051	0.000	0.000	0.000	0.000	0.000	0.000	0.028	0.094
Kluyveromyces	0.006	0.035	0.000	0.000	0.009	0.042	0.000	0.000	0.000	0.000	0.000	0.000
Ustilaginales_unidentified_1	0.000	0.000	0.005	0.034	0.000	0.000	0.000	0.000	0.008	0.040	0.000	0.000
Golovinomyces	0.003	0.018	0.000	0.000	0.004	0.021	0.000	0.000	0.000	0.000	0.000	0.000
Pezizomycetes_unidentified_1	0.003	0.018	0.000	0.000	0.004	0.021	0.000	0.000	0.000	0.000	0.000	0.000
Knufia	0.000	0.000	0.003	0.017	0.000	0.000	0.000	0.000	0.000	0.000	0.009	0.031
Dipodascaceae_unidentified	0.000	0.000	0.003	0.017	0.000	0.000	0.000	0.000	0.000	0.000	0.009	0.031
Wickerhamomyces	0.000	0.000	0.003	0.017	0.000	0.000	0.000	0.000	0.000	0.000	0.009	0.031
Schizosaccharomyces	0.000	0.000	0.003	0.017	0.000	0.000	0.000	0.000	0.004	0.020	0.000	0.000
Coniochaeta	0.000	0.000	0.003	0.017	0.000	0.000	0.000	0.000	0.004	0.020	0.000	0.000
Sordariaceae_unidentified	0.000	0.000	0.003	0.017	0.000	0.000	0.000	0.000	0.004	0.020	0.000	0.000
