# Supplementary material for: New evidences on the altered gut microbiota in autism spectrum disorders
Source: Microbiome. 2017 Feb 22;5:24. doi: 10.1186/s40168-017-0242-1 (PMC5320696; doi:10.1186/s40168-017-0242-1)
Supplement: Additional file 11: Table S8. — Spearman’s correlation analysis among the most abundant bacterial genera and fungal genera in autistic and neurotypical subjects. (PDF 253 kb) [file 40168_2017_242_MOESM11_ESM.pdf]

Supplementary Table 8: Spearman's correlation analysis among the most abundant bacterial genera and fungal genera in autistic and neurotypical subjects.

**Autistic subjects**	**Spearman's *r* correlations**				**FDR-corrected *p*-values**			
Taxa	*Aspergillus*	*Candida*	*Penicillium*	*Malassezia*	*Aspergillus*	*Candida*	*Penicillium*	*Malassezia*
Bifidobacterium	-0.145	-0.021	0.047	0.029	0.945	0.996	0.996	0.996
Bacteroides	0.213	-0.109	-0.143	0.048	0.945	0.945	0.945	0.996
Faecalibacterium	-0.037	0.143	-0.079	-0.140	0.996	0.945	0.996	0.945
Blautia	-0.110	0.121	0.241	-0.011	0.945	0.945	0.945	0.996
Ruminococcus	0.072	-0.167	0.174	0.044	0.996	0.945	0.945	0.996
Clostridium XI	0.098	-0.129	0.230	-0.001	0.970	0.945	0.945	0.996
Streptococcus	-0.050	0.144	0.178	-0.109	0.996	0.945	0.945	0.945
Gemmiger	0.007	0.174	-0.026	-0.391	0.996	0.945	0.996	0.645
Lachnospiracea incertae sedis	-0.165	0.301	0.013	-0.235	0.945	0.941	0.996	0.945
Escherichia/Shigella	-0.094	0.110	-0.008	0.136	0.976	0.945	0.996	0.945
Alistipes	0.147	-0.205	-0.111	0.130	0.945	0.945	0.945	0.945
Anaerostipes	-0.041	0.277	-0.211	-0.162	0.996	0.941	0.945	0.945
Clostridium XVIII	-0.139	0.167	-0.145	-0.108	0.945	0.945	0.945	0.945
Dorea	0.089	0.009	-0.024	0.023	0.996	0.996	0.996	0.996
Collinsella	0.059	-0.054	0.301	0.193	0.996	0.996	0.941	0.945
Clostridium sensu stricto	0.111	0.100	0.187	-0.139	0.945	0.970	0.945	0.945
Dialister	0.156	-0.107	-0.050	0.058	0.945	0.945	0.996	0.996
Erysipelotrichaceae incertae sedis	0.009	-0.290	0.015	0.018	0.996	0.941	0.996	0.996
Coprococcus	-0.149	-0.004	-0.171	-0.250	0.945	0.996	0.945	0.945
Clostridium IV	0.302	-0.231	-0.003	0.020	0.941	0.945	0.996	0.996
Lactobacillus	0.062	0.045	-0.002	0.009	0.996	0.996	0.996	0.996
Turicibacter	0.334	-0.334	0.425	0.417	0.941	0.941	0.606	0.606
Oscillibacter	0.225	-0.281	-0.065	0.246	0.945	0.941	0.996	0.945
Clostridium XlVa	0.154	-0.018	-0.038	-0.002	0.945	0.996	0.996	0.996

**Neutotypical subjects**	**Spearman's *r* correlations**				**FDR-corrected *p*-values**			
Taxa	*Aspergillus*	*Candida*	*Penicillium*	*Malassezia*	*Aspergillus*	*Candida*	*Penicillium*	*Malassezia*
Bifidobacterium	0.617	-0.097	-0.060	0.170	0.004	0.821	0.909	0.716
Bacteroides	-0.231	-0.049	0.229	-0.307	0.690	0.947	0.690	0.592
Faecalibacterium	-0.324	0.014	0.238	-0.254	0.592	0.978	0.690	0.690
Blautia	-0.111	0.207	-0.181	0.163	0.783	0.690	0.690	0.716
Ruminococcus	-0.321	-0.127	0.112	-0.069	0.592	0.774	0.783	0.903
Clostridium XI	0.154	0.055	0.185	0.004	0.725	0.927	0.690	0.983
Streptococcus	-0.010	-0.072	0.037	0.198	0.982	0.903	0.956	0.690
Gemmiger	-0.196	0.091	0.247	0.005	0.690	0.843	0.690	0.983
Lachnospiracea incertae sedis	-0.236	-0.024	-0.065	-0.166	0.690	0.978	0.903	0.716
Escherichia/Shigella	0.141	0.389	-0.085	0.143	0.747	0.378	0.865	0.747
Alistipes	-0.209	-0.004	-0.306	-0.152	0.690	0.983	0.592	0.725
Anaerostipes	-0.041	0.180	-0.273	0.016	0.956	0.690	0.690	0.978
Clostridium XVIII	0.111	0.163	-0.158	-0.206	0.783	0.716	0.716	0.690
Dorea	0.071	0.071	0.101	0.037	0.903	0.903	0.817	0.956
Collinsella	-0.116	0.210	0.019	0.123	0.783	0.690	0.978	0.774
Clostridium sensu stricto	0.099	0.066	0.216	0.127	0.820	0.903	0.690	0.774
Dialister	-0.255	-0.158	0.123	0.030	0.690	0.716	0.774	0.967
Erysipelotrichaceae incertae sedis	0.264	0.043	-0.205	0.208	0.690	0.956	0.690	0.690
Coprococcus	0.128	0.106	-0.184	0.188	0.774	0.802	0.690	0.690
Clostridium IV	-0.189	-0.014	-0.060	-0.188	0.690	0.978	0.909	0.690
Lactobacillus	0.142	0.121	-0.397	0.299	0.747	0.774	0.378	0.596
Turicibacter	0.037	0.021	0.353	-0.215	0.956	0.978	0.573	0.690
Oscillibacter	-0.317	-0.159	-0.127	-0.208	0.592	0.716	0.774	0.690
Clostridium XlVa	-0.270	-0.032	0.013	-0.415	0.690	0.967	0.978	0.378
